# Evaluation of Postoperative Pain Frequency in Single‐Session Endodontic Treatments With Patency and Foraminal Enlargement

**DOI:** 10.1002/cre2.70313

**Published:** 2026-02-23

**Authors:** Viviane Barbosa Godoy, Ana Grasiela Limoeiro, Vanessa Sandini, Vini Mehta, Wayne Martins Nascimento, Marilia Fagury Videira Marceliano‐Alves, Marcos Frozoni

**Affiliations:** ^1^ Department of Endodontics São Leopoldo Mandic School and Research Institute Campinas Brazil; ^2^ Department of Dentistry, Endodontics and Dental Materials Bauru School of Dentistry University of São Paulo (USP) Bauru Brazil; ^3^ University of Vale do Itajaí Itajaí Brazil; ^4^ Faculty of Dentistry University of Ibn al‐Nafis for Medical Sciences Sana'a Yemen; ^5^ Department of Dental Research Cell Dr. D. Y. Patil Dental College & Hospital Dr. D. Y. Patil Vidyapeeth (Deemed to be University) Pune India; ^6^ Postgraduate Program in Dentistry Iguaçu University Nova Iguaçu Brazil; ^7^ Department of Endodontics Maurício de Nassau University Centre (UNINASSAU) Rio de Janeiro Brazil

**Keywords:** apical foramen, endodontics, postoperative pain, root canal treatment, single‐session treatment

## Abstract

**Objective:**

This study aimed to evaluate the incidence of postoperative pain in single‐session endodontic treatments with foraminal instrumentation, considering factors such as pulpal diagnosis, tooth position, type of endodontic sealer, and sealer extrusion.

**Materials and Methods:**

Treatments were performed on 153 teeth of patients aged 12–80 years diagnosed with asymptomatic irreversible pulpitis or asymptomatic pulpal necrosis with or without periapical lesions. The obturation sealers used were AH Plus, Endomethasone‐N, and Sealer Plus. Postoperative pain intensity was recorded by patients using a modified numerical rating scale (NRS) ranging from 0 (no pain) to 10 (severe pain).

**Results:**

No statistically significant differences in pain scores were found at 12, 24, or 48 h in relation to gender, age, tooth position, pulpal diagnosis, type of sealer, or extrusion (*p* > 0.05). The most common pain levels classified as uncomfortable were observed at 12 and 24 h, while 94.1% of patients reported no pain at 48 h; the remaining pain was mild. Pain scores decreased significantly after 48 h for all variables.

**Conclusion:**

Single‐session endodontic treatment with foraminal instrumentation showed a low incidence of postoperative pain that was not influenced by the variables analyzed in this study.

**Null Hypothesis:**

The null hypothesis was that there was no association between the occurrence of postoperative pain and (i) age, (ii) patient gender, (iii) pulp diagnosis, (iv) tooth position, (v) endodontic sealer type, and (vi) sealer extrusion.

## 1 Introduction

Pain and discomfort after endodontic procedures are undesirable for both dentists and patients, making management crucial (El Mubarak et al. 2010). Reports indicate that post‐treatment pain occurs in 3%–58% of cases and is influenced by individual and procedural variables (Ferreira et al. 2020). Causes of postoperative pain include mechanical, chemical or microbiological injury to the dental tissue (Graunaite et al. 2018). Cleaning and shaping of the foraminal area aim to disinfect the root canal system and promote periapical healing (Saini et al. 2016). However, foraminal instrumentation has been associated with increased pain shortly after endodontic therapy (Borges Silva et al. 2017), although some studies have found no such association (de Oliveira Escócio et al. 2023; da Silva et al. 2023). Additional factors such as patient age and gender, tooth type, pre‐existing pain, pulp and periapical condition, occlusal contact and apical radiolucency may influence postoperative symptoms (de Freitas Portela et al. 2021). Endodontic sealers can reach periradicular tissues through the apical foramen and lateral canals, potentially causing local inflammatory processes that lead to postoperative pain (Graunaite et al. 2018).

Endodontic sealers must be biocompatible due to the prolonged exposure to periapical tissue (Silva et al. 2022). Zinc oxide and eugenol‐based sealers are commonly used (Komabayashi et al. 2020), with zinc oxide providing antimicrobial effects (Monteiro et al. 2023), and eugenol offering antimicrobial, anti‐inflammatory, and antioxidant properties (Jeanneau et al. 2020).

The selection of AH Plus, Endomethasone‐N, and Sealer Plus endodontic sealers for this study was based on their distinct compositions and properties, reflecting a representation of materials widely used in endodontic practice and allowing for the evaluation of different obturation approaches. AH Plus (Dentsply Maillefer, Ballaigues, Switzerland), an epoxy resin‐based sealer, is valued for its long‐term dimensional stability, low solubility, effective apical sealing, and dentin micro‐retention, although in vitro studies have indicated mild cytotoxicity and the release of toxic monomers such as bisphenol A diglycidyl ether (Vertuan et al. 2018; Lodienė et al. 2013). In contrast, Endomethasone‐N (Septodont, Saint‐Maur‐des‐Fossés, Cedex, France), a zinc oxide‐eugenol based sealer, incorporates hydrocortisone, a component intended to modulate inflammation and potentially contribute to the reduction of postoperative pain (Jeanneau et al. 2020). Conversely, Sealer Plus (MK Life, Porto Alegre, Brazil), while having a similar composition to AH Plus, is distinguished by the addition of calcium hydroxide in both the base and catalyst, imparting the ability to promote an alkaline pH, antibacterial activity, and accelerate tissue repair (Cintra et al. 2017).

A comprehensive approach to post‐endodontic pain factors reflects the clinical reality in which different factors interact (Arias et al. 2013). The aim of this study was to investigate the occurrence of postoperative pain in single‐session endodontic treatments with foraminal instrumentation, considering gender, age, pulp diagnosis, tooth position, type of endodontic sealer and sealer extrusion over 12, 24, and 48 h. The null hypothesis was that there was no association between the occurrence of postoperative pain and (i) age, (ii) patient gender, (iii) pulp diagnosis, (iv) tooth position, (v) endodontic sealer type, and (vi) sealer extrusion.

## 2 Materials and Methods

This retrospective clinical study was approved by the local research ethics committee (CAAE: 75536123.4.0000.5374). Each patient signed an informed consent form. The endodontic treatments were performed in a single session with patency and foraminal enlargement between 2017 and 2018. The dependent variable of the study was the intensity of postoperative pain in endodontic treatments performed in a single session with foraminal instrumentation. The study was carried out in adherence to the Preferred Reporting Items for Observational studies in Endodontics (PROBE) (Supporting Information File 1).

The study included 153 teeth from patients aged 12–80 years selected based on the following criteria: individuals aged 12–80 years requiring single‐session endodontic treatment for teeth diagnosed with either asymptomatic irreversible pulpitis or asymptomatic pulpal necrosis, with or without periapical lesions. Eligible teeth included anterior or posterior permanent teeth exhibiting a fully formed dental apex. Furthermore, all participants were required to be generally healthy and confirmed not to have used any analgesics, anti‐inflammatory drugs, or antibiotics prior to the commencement of treatment. Exclusion criteria included symptomatic teeth, open apex, resorption, previous endodontic treatment, calcified canals, inability to achieve patency, perforations, broken instruments, multi‐session treatments, pregnancy, or use of medications affecting pain perception or post‐treatment pain analysis.

The diagnosis was based on symptoms described by the patient, such as spontaneous pain, and responses to thermal tests conducted with cold spray and heated gutta‐percha. Percussion and palpation tests were used to detect painful reactions, and a radiographic examination was performed in accordance with AAE Consensus Conference on Diagnostic Terminology (Glickman 2009).

Teeth were categorized as anterior/posterior and maxillary/mandibular for the analysis of pain frequency according to tooth position.

### 2.1 Description of the Root Canal Preparation Technique

All procedures were performed in a single session by four endodontists with more than 6 years of experience. Patients received local anesthesia with 2% lidocaine with 1:100,000 epinephrine (DFL, Rio de Janeiro, Brazil). Caries and restorations were removed, and a standard access cavity was prepared. The root canals were isolated with rubber dam in all cases. The canals were explored with C‐Pilot files #10 and #15 (VDW, Munich, Germany). Irrigation with 1 mL of 2% chlorhexidine gel using a syringe (20 mm × 0.55 mm needle) was performed before the use of each subsequent file. Chemo mechanical shaping of the cervical and middle thirds was performed with Logic 25/.05, 30/.05, or 35/.05 rotary files (Bassi/Easy, Belo Horizonte, Minas Gerais, Brazil), depending on the canal diameter. The apical third was negotiated with a C‐Pilot file #10 (VDW, Munich, Germany) until patency was achieved, which was confirmed using an electronic apex locator. The working length (WL) was determined using an electronic apex locator by advancing a C‐Pilot file until patency was achieved and setting the WL at the point corresponding to the “zero” mark on the apex locator display.

Root canal shaping was performed using Logic rotary instruments (Bassi/Easy): 30/.05 for narrow canals (when the initial apical file was #15), and 35/.05 or 40/.05 for wide canals (when the initial apical file was #25). The instruments were advanced into the root canal using a pecking motion with a maximum amplitude of 3 mm per movement until the WL defined for each tooth was reached. At the end of the preparation, the root canals were irrigated with 5 mL of 2% chlorhexidine solution and 5 mL of 0.9% saline solution for final rinse. Final irrigation was performed with 1 mL of 17% EDTA, activated using the Easy Clean system (Bassi/Easy) with a rotating motion.

### 2.2 Obturation of the Root Canal

**Chart 1:**
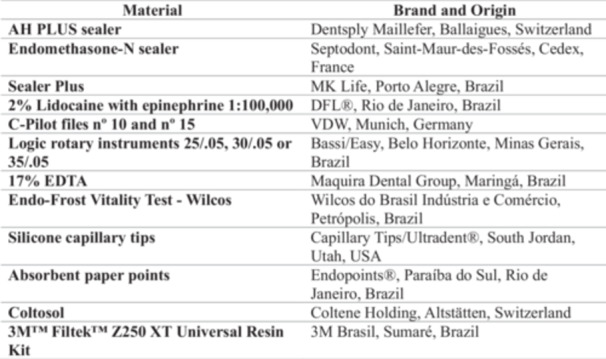
Materials used in the study and their respective brand and origin.

Root canals were dried using silicone capillary tips (Capillary Tips/Ultradent, South Jordan, Utah, USA) and absorbent paper points (Endopoints, Paraíba do Sul, Rio de Janeiro, Brazil) that were matched to the master apical file size and inserted to the WL. The endodontic sealers used included AH Plus (Dentsply Maillefer), Endomethasone‐N (Septodont), and Sealer Plus (MK Life), which were applied to the root canal walls or the master gutta‐percha cone prior to obturation. The obturation technique involved the use of a single gutta‐percha cone, potentially thermoplasticized, followed by hydraulic compaction using Schilder's condensers. The coronal 3 mm of the root canal was sealed with Coltosol (Vigodent), and the coronal access cavity was restored with composite resin. Occlusal adjustment was performed, followed by a final digital periapical radiograph. The presence of inadvertent extrusion of the endodontic sealer was assessed by an endodontist on all final radiographs of the treated teeth using the ruler function of the SOPRO Imaging 2.41 software (Micro Imagem, Rio de Janeiro, Brazil), by two calibrated Endodontic specialists, and in case of disagreement, a third specialist acted as a tie‐breaker. All materials and their respective origin and brand are available in Chart 1.

### 2.3 Postoperative Pain Assessment

After endodontic treatment, patients used a modified numerical rating scale (NRS) (Ferreira‐Valente et al. 2011) with scores ranging from 0 to 10, where zero represented no pain and 10 represented severe pain, to assess postoperative pain at three time points: 12, 24, and 48 h. The scale was graded as follows: 0: No pain (the patient feels fine); 1–3: Mild pain (the patient can distract themselves, does not need painkillers); 4–6: Moderate pain (the patient feels moderate pain even when concentrating on something else, needs painkillers); 7–9: Severe pain (the patient cannot perform any other activity, must lie down to rest; analgesics provide little to no relief) and, 10: Worst possible pain (the patient cannot sleep and suffers from psychological stress).

After 48 h, all patients were contacted by telephone by an endodontist from the team to report their pain scores at the different time points. Patients reporting moderate pain (scores 4–6) were advised to take analgesics for pain control. The drug of choice was Dipyrone 500 mg, with a recommended dosage of 1 tablet every 6 h for up to 3 days.

### 2.4 Statistical Analysis

Gender, age range, tooth position, endodontic diagnosis, type of sealer used, and whether extrusion occurred were reported using absolute (*n*) and relative (%) frequencies. The pain reported by the participants 12, 24, and 48 h after treatment was also described. Mann–Whitney or Kruskal–Wallis tests were used to determine whether gender, age range, tooth position, endodontic diagnosis, type of sealer, and its extrusion influenced pain scores. Friedman tests were used to compare pain scores over time. Statistical calculations were performed using SPSS 23 (SPSS INC., Chicago, IL, USA) and BioEstat 5.0 (Fundação Mamirauá, Belém, PA, Brazil), with a significance level of *α* = 0.05.

## 3 Results

As the treatment was performed in a single session and patients were contacted by telephone, there was no sample loss, so all 153 patients remained in the study. Most of the 153 teeth included in this study were from female participants. The highest proportion of participants was in the 36‐ to 50‐year‐old age group, followed by the over‐50s and the 18‐ to 35‐year‐olds (Table 1). Regarding the dental arch, 55.6% of the teeth were in the maxillary arch and the remaining 44.4% in the mandibular arch. The sample included predominantly posterior teeth (83.0%) compared to anterior teeth (17.0%). Teeth located in the mandibular posterior (41.8%) and maxillary posterior (41.2%) regions were the most common categories when considering both arch and region, as shown in Table 1.

**Table 1:** Absolute (*n*) and relative (%) frequencies of gender, age group, dental arch, region, diagnosis, filling cement, and its leakage from the teeth included in the study.

Characterization of the sample	*n*	%
Gender		
Male	54	35.3
Female	99	64.7
Age range		
< 18 years	17	10.5
18–35 years	40	26.1
35–50 years	54	35.3
> 50 years	42	28.1
Dental arch		
Maxilary	77	55.6
Mandibular	76	44.4
Region in the arch		
Anterior	26	17.0
Posterior	127	83.0
Arch/region		
Superior/anterior	22	14.4
Superior/posterior	63	41.2
Inferior/anterior	4	2.6
Inferior/posterior	65	41.8
Diagnosis		
Asymptomatic pulpitis	31	20.3
Necrosis without lesion	59	38.6
Necrosis with lesion	63	41.2
Obturator cements		
Endomethazone	71	46.4
AH Plus	50	32.7
Sealer Plus	32	20.9
Sealer extrusion		
No		49.0
Yes	78	51.0

The distribution of pulpal diagnoses was asymptomatic irreversible pulpitis (20.3%), pulp necrosis without periapical lesions (38.6%), and pulp necrosis with periapical lesions (41.2%) (Table 1). Endomethasone was the most used sealer (46.4%), followed by AH Plus (32.7%) and Sealer Plus (20.9%). The proportion of cases with sealer extrusion was 51.0% (Table 1). Regardless of the time point, the most common pain score reported was 0 (Table 2).

**Table 2:** Absolute (*n*) and relative (%) frequencies of pain scores (0–10) reported by participants 12, 24, and 48 h after endodontic filling.

Pain score	12 h	24 h	48 h
0	106 (69.3%)	125 (81.7%)	144 (94.1%)
1	8 (5.2%)	6 (3.9%)	1 (0.7%)
2	7 (4.6%)	6 (3.9%)	2 (2.0%)
3	6 (3.9%)	4 (2.6%)	0 (0.0%)
4	4 (2.6%)	3 (2.0%)	0 (0.0%)
5	4 (2.6%)	4 (2.6%)	2 (1.3%)
6	4 (2.6%)	4 (2.6%)	3 (2.0%)
7	5 (3.3%)	0 (0.0%)	0 (0.0%)
8	5 (3.3%)	1 (0.7%)	0 (0.0%)
9	2 (1.3%)	0 (0.0%)	0 (0.0%)
10	3 (2.0%)	0 (0.0%)	0 (0.0%)

Table 3 presents the frequency distribution of participants reporting no pain and those with pain rated as mild, moderate, severe, and unbearable. This shows that pain at 12 and 24 h after endodontic obturation was most often classified as mild. After 48 h, the proportion of patients reporting no pain increased to 94.1%, and the remaining cases with residual pain were classified as mild (2.6%) or moderate (3.3%).

**Table 3:** Absolute (*n*) and relative (%) frequencies of pain classification reported by participants 12, 24, and 48 h after endodontic filling.

Pain classification	12 h	24 h	48 h
Absent	106 (69.3%)	125 (81.7%)	144 (94.1%)
Discomfort	21 (13.7%)	16 (10.5%)	4 (2.6%)
Moderate	11 (7.2%)	11 (7.2%)	5 (3.3%)
Strong	12 (7.8%)	1 (0.7%)	0 (0.0%)
Intense	3 (2.0%)	0 (0.0%)	0 (0.0%)

No statistically significant differences in pain scores were found between male and female participants after 12, 24, or 48 h. There were also no significant differences in pain scores when comparing participants aged < 18, 18–35, 36–50, and > 50 years. Pain scores were not influenced by tooth position (maxilla or mandible, anterior or posterior). The pulpal diagnosis (asymptomatic irreversible pulpitis or pulp necrosis with or without periapical lesions) did not significantly influence postoperative pain scores at any of the three evaluation time points (12, 24, and 48 h). The type of endodontic sealer used and whether extrusion occurred also had no significant effect on postoperative pain scores (Table 4).

**Table 4:** Medians and minimum and maximum values of pain reported by the participants, according to the independent variable and time of assessment.

Independent variable	Evaluation moment			*p* value
	12 h	24 h	48 h	
Gender				
Male	0 (0; 8) Ab	0 (0; 3) Aab	0 (0; 0) Aa	*p* = 0.037
Female	0 (0; 10) Ab	0 (0; 8) Aab	0 (0; 6) Aa	*p* = 0.002
	*p* = 0.390	*p* = 0.202	*p* = 0.354	
Age group				
< 18 years	0 (0; 8) Aa	0 (0; 2) Aa	0 (0; 0) Aa	*p* = 0.646
18–35 years	0 (0; 8) Aa	0 (0; 6) Aa	0 (0; 6) Aa	*p* = 0.131
36–50 years	0 (0; 10) Ab	0 (0; 8) Aab	0 (0; 5) Ab	*p* = 0.002
> 50 years	0 (0; 7) Aa	0 (0; 6) Aa	0 (0; 6) Aa	*p* = 0.184
	*p* = 0.303	*p* = 0.461	*p* = 0.950	
Tooth position				
Maxillary/anterior	0 (0; 10) Aa	0 (0; 8) Aa	0 (0; 2) Aa	*p* = 0.554
Maxillary/posterior	0 (0; 10) Ab	0 (0; 6) Aab	0 (0; 5) Ab	*p* = 0.008
Mandibular/anterior	0 (0; 6) Aa	0 (0; 4) Aa	0 (0; 0) Aa	*p* = 0.779
Mandibular/posterior	0 (0; 8) Ab	0 (0; 6) Aab	0 (0; 6) Aa	*p* = 0.013
	*p* = 0.761	*p* = 0.864	*p* = 0.996	
Diagnosis				
Asymptomatic pulpitis	0 (0; 10) Ab	0 (0; 3) Aab	0 (0; 0) Aa	*p* = 0.017
Necrosis without lesion	0 (0; 10) Ab	0 (0; 8) Aab	0 (0; 6) Aa	*p* = 0.017
Necrosis with lesion	0 (0; 10) Ab	0 (0; 6) Aab	0 (0; 5) Aa	*p* = 0.033
	*p* = 0.800	*p* = 0.580	*p* = 0.578	
Cement				
Endomethazone	0 (0; 10) Ab	0 (0; 6) Aab	0 (0; 6) Aa	*p* = 0.041
AH Plus	0 (0; 9) Ab	0 (0; 8) Aab	0 (0; 6) Aa	*p* = 0.005
Sealer Plus	0 (0; 10) Aa	0 (0; 6) Aa	0 (0; 6) Aa	*p* = 0.208
	*p* = 0.426	*p* = 0.642	*p* = 0.799	
Leakage				
No	0 (0; 10) Ab	0 (0; 8) Aab	0 (0; 6) Aa	*p* = 0.008
Yes	0 (0; 10) Ab	0 (0; 6) Aab	0 (0; 6) Aa	*p* = 0.008
	*p* = 0.891	*p* = 0.903	*p* = 0.661	

*Note:* Uppercase and lowercase letters represent the differences found by the Mann–Whitney, Kruskal–Wallis, and Friedman tests (*α* = 0.05), based on the mean orders. Different capital letters indicate differences within each column, considering each independent variable. Different lowercase letters indicate a difference within each row, considering each independent variable.

Both men and women experienced significantly less pain at 48 h compared to the 12‐h assessment. Pain scores at 24 h were intermediate and did not differ significantly from the scores at 12 or 48 h. This pattern of pain reduction over time (significantly less pain at 48 h compared to 12 h) was also observed for participants aged 36–50 years and for teeth located in the posterior mandible and maxilla. Regardless of the pulpal diagnosis (asymptomatic irreversible pulpitis or pulp necrosis with or without a periapical lesion), pain scores were significantly lower at 48 h compared to 12 h. This pattern was also observed when using AH Plus and Endomethasone sealers. Regardless of whether endodontic sealer extrusion occurred, pain scores were also significantly lower at 48 h compared to 12 h (Table 4).

In participants aged < 35 years and > 50 years, pain scores showed no statistically significant differences when comparing the time points (12, 24, and 48 h). The same result (no significant difference in pain scores over time) was observed for teeth in the anterior region of the maxilla and mandible and when Sealer Plus was used for obturation (Table 4).

## 4 Discussion

In this study, the incidence of postoperative pain after endodontic treatments performed in a single session with foraminal instrumentation was evaluated at 12, 24, and 48 h. This evaluation considered variables such as gender, age, pulpal diagnosis, tooth position, type of endodontic sealer, and sealer extrusion. The null hypothesis of this study was that there is no association between the occurrence of postoperative pain and (i) age, (ii) patient gender, (iii) pulpal diagnosis, (iv) tooth position, (v) type of endodontic sealer, and (vi) sealer extrusion.

Randomized clinical trials have investigated the effect of foraminal enlargement on postoperative pain. One study found no difference in pain levels between groups with and without foraminal enlargement when using hand files (Silva et al. 2013). Cruz Junior et al. (2016) reported more pain at 24 h with foraminal enlargement using a reciprocating system, and Yaylali et al. (2017) found more severe pain in the first 2 days in molars using a rotary system.

This study reported no postoperative pain in 69.3% of patients within the first 12 h, 81.7% after 24 h, and 94.1% after 48 h, which aligns with the findings of Demenech et al. (2021). In cases where pain was reported, it was primarily categorized as mild discomfort, with patients reporting pain scores between 1 and 3 (mild intensity, allowing for distraction, and not requiring analgesics). This pain decreased over time, as observed in other studies (Çiçek et al. 2017; Comparin et al. 2017). This low symptom rate was attributed to treatment parameters, including the crown‐down technique and the use of 2% chlorhexidine gel, which helps minimize debris extrusion (Arruda‐Vasconcelos et al. 2019).

The decision to focus exclusively on asymptomatic patients, with diagnoses of asymptomatic irreversible pulpitis or pulpal necrosis with or without periapical lesions, represents a crucial methodological distinction of this study. This selection of patients allowed for a focused evaluation of factors influencing postoperative pain in the absence of pre‐existing discomfort. The consistent finding in endodontic literature, also acknowledged in the manuscript's discussion, is that preoperative pain is a strong predictor of postoperative pain (Turkyilmaz et al. 2024). This rigorous patient selection is, therefore, a key factor contributing to the low incidence of postoperative pain observed in this investigation, with 69.3% of patients reporting no pain at 12 h and 94.1% at 48 h. This result aligns with expectations for treatments performed on asymptomatic teeth, where the initial inflammatory burden is generally lower. Studies investigating postoperative pain specifically in asymptomatic cases frequently report similarly low rates of discomfort (Ferreira et al. 2020; Turkyilmaz et al. 2024).

Consequently, although the findings regarding the lack of significant influence of variables such as gender, age, tooth position, pulpal diagnosis, sealer type, and sealer extrusion on postoperative pain are valuable for understanding the results in this specific context, direct comparisons with studies that include a mix of symptomatic and asymptomatic cases should be approached with caution.

Consistent with previous studies (Ferreira et al. 2020; Koçer et al. 2022), no significant difference in postoperative pain was found between genders, thus supporting the first null hypothesis. A systematic review concluded that research has not provided clear and consistent results regarding gender and pain sensitivity (Racine et al. 2012). This suggests that differences in pain sensitivity between genders may be related to other variables such as reproductive status, age, and the presence of disease (Pieretti et al. 2016) implying that gender is not directly related to postoperative endodontic pain.

In this study, age also had no significant influence on the occurrence of postoperative signs and symptoms, which is consistent with other studies (AlRahabi 2017; Machado et al. 2021) Therefore, the second null hypothesis was also supported. However, some authors (Arias et al. 2013) have argued that older patients experienced more postoperative pain than younger patients, possibly due to factors such as lower pain tolerance, longer treatment duration resulting from severe calcifications, reduced blood flow, and delayed healing (Arias et al. 2013; Machado et al. 2021). Conversely, another study (Ehrmann et al. 2007) described that younger patients expected more severe pain, although there is no definitive evidence that increasing age is associated with a progressive loss of nociceptive sensitivity (Walco 2004).

Previous studies have shown that the presence of preoperative pain is a strong predictor of postoperative pain (Graunaite et al. 2018; Comparin et al. 2017). For this reason, only asymptomatic patients were included in this study. The diagnosis of asymptomatic pulpitis or pulp necrosis, with or without a periapical lesion, did not have a significant impact on postoperative pain scores at any of the three assessment time points (12, 24, and 48 h), thus supporting the third null hypothesis. Furthermore, the presence of periapical lesions in cases of pulpal necrosis was not associated with postoperative pain in this study, confirming findings from previous studies (Demenech et al. 2021; Machado et al. 2021).

Post‐endodontic pain is influenced by patient‐ and tooth‐specific factors (Gomes et al. 2017). In the present study, tooth position (maxilla/mandible/anterior/posterior) did not show a statistically significant difference in pain levels, which is consistent with other studies (Demenech et al. 2021; Machado et al. 2022) and supports the fourth null hypothesis. Conversely, a higher level of post‐endodontic pain was significantly associated with posterior teeth, with the mandibular premolars having higher pain indices than the upper anterior teeth and in the mandibular arch (Graunaite et al. 2018). The potentially longer duration of the procedure for posterior teeth could also partially explain such results (Demenech et al. 2021).

Despite the different compositions and potential interactions between the sealers and periapical tissues, no significant difference in post‐treatment pain was observed among the endodontic sealers (AH Plus, Endomethasone‐N, or Sealer Plus) tested at all time points. This finding is consistent with other studies (Graunaite et al. 2018; Ferreira et al. 2020; Tan et al. 2021; Drumond et al. 2021) and supports the fifth null hypothesis. However, significant decreases in pain scores from the 12‐h to the 48‐h interval were observed *within* the AH Plus and Endomethasone groups. According to Aslan and Dönmez Özkan (2021), minor extrusion of the sealer may not cause pain due to the small contact area with the periapical tissue. Additionally, under clinical conditions, the concentration of potentially toxic substances can be reduced by tissue fluid (Okiji and Yoshiba 2009).

When comparing the occurrence of postoperative pain with the extrusion of endodontic sealer, there was no statistical difference between the groups with and without extrusion, which supports the sixth null hypothesis. These results suggest that sealer extrusion, in small amounts, is not necessarily associated with pain, consistent with other clinical studies where AH Plus was used as a sealer 6,8. Minor extrusion of endodontic filling materials is generally considered acceptable if no important anatomical structures are affected (Shashirekha et al. 2018).

In endodontic postoperative cases, approximately 47% of patients report moderate to severe pain, with a higher incidence typically observed between 6 and 24 h (Shamszadeh et al. 2021). The time intervals chosen for this study (12, 24, and 48 h) capture the period following the initial peak of postoperative pain (often 6–8 h) and encompass the expected peak of inflammation (between 36 and 48 h), allowing evaluation beyond the immediate post‐procedure period.

In this study, 2% chlorhexidine gel was used as the chemical irrigant due to its biocompatibility with periapical tissue (Gomes et al. 2017) and its association with a high success rate in endodontic procedures (da Silva et al. 2023).

The Logic rotary system was selected in this study, likely based on dental anatomy considerations. A previous study (Çiçek et al. 2017) investigated the influence of instrument kinematics (rotational or reciprocating) on postoperative pain and concluded that the type of movement did not influence the results.

Finally, in the present study, postoperative pain was significantly reduced 48 h after endodontic treatment compared to the initial assessment at 12 h, confirming findings from other studies (de Oliveira Escócio et al. 2023; Pak and White 2011).

The study had several limitations, including a lack of standardization regarding the specific instruments used within the system, the type of sealer used in each tooth (as multiple were included), and the individual postgraduate student performing the procedure. Postoperative pain data relied on patient self‐reports, which can be influenced by subjective and emotional factors, as pain perception varies widely among individuals. Further research is needed on the long‐term effects of foraminal enlargement, potentially through observational cohort studies, and its overall impact on postoperative pain.

The study exhibited significant strengths, primarily its rigorous patient selection that exclusively focused on asymptomatic individuals, which is a methodological distinction for isolating the true effects on postoperative pain. Pain intensity was reliably measured using a modified NRS, a tool widely validated for such assessments as supported by previous research (Ferreira‐Valente et al. 2011), which revealed a low incidence of postoperative pain, consistent with observations from other study (Demenech et al. 2021). The comprehensive analysis of numerous variables—including gender, age, and different endodontic sealers—provided robust insights into their non‐association with pain, often confirming earlier findings (Ferreira‐Valente et al. 2011). Furthermore, the complete retention of all 153 patients throughout the study, achieved through diligent telephone follow‐up, significantly bolstered the integrity and completeness of the valuable dataset.

Given the limitations of the study, such as the lack of standardization for specific instruments and the inter operator variability performing procedures, further clinical studies are essential to validate and generalize these findings. Moreover, relying solely on subjective patient self‐reports for pain assessment highlights the need for future research to incorporate more objective measures or blinded designs. Finally, long‐term observational cohort studies are necessary to investigate the effects of foraminal enlargement and its impact on postoperative pain over extended periods.

## 5 Conclusion

This study found that single‐session endodontic treatment with foraminal instrumentation had a low incidence of postoperative pain, which significantly decreased within the first 48 h. None of the variables studied (gender, age, pulpal diagnosis, tooth position, type of endodontic sealer, or sealer extrusion) were found to significantly influence the incidence or intensity of postoperative pain.

## Author Contributions

All authors contributed to the study conceptualization and design. Material preparation, data collection, and analysis were performed by Viviane Barbosa Godoy, Ana Grasiela Limoeiro, Marilia Fagury Videira Marceliano‐Alves. The manuscript was written and revised by Vanessa Sandini, Vini Mehta, Wayne Martins Nascimento, Marilia Fagury Videira Marceliano‐Alves, and Marcos Frozoni. Wayne Martins Nascimento prepared the figures. All authors commented on previous versions of the manuscript. All authors read and approved the final manuscript.

## Ethics Statement

As this study involves extracted human teeth, it received approval from the São Leopoldo Mandic Research Ethics Committee under protocol number CAAE 75536123.4.0000.5374.

## Consent

The authors consent the journal to publish the current manuscript.

## Conflicts of Interest

The authors declare no conflicts of interest.

## Supporting information

PROBE_2023_checklist.

## Data Availability

The data that support the findings of this study are available on request from the corresponding author.
